# Perceptions of Pharmacy Involvement in Social Prescribing Pathways in England, Scotland and Wales

**DOI:** 10.3390/pharmacy7010024

**Published:** 2019-03-04

**Authors:** Denise A. Taylor, Gina M. Nicholls, Andrea D.J. Taylor

**Affiliations:** 1 Graduate School of Nursing, Midwifery and Health, Faculty of Health, Victoria University of Wellington, P.O. Box 7625, Newtown, Wellington 6242, New Zealand; 2 Department of Pharmacy and Pharmacology, University of Bath, Bath BA2 7AY, UK; gnic447@aucklanduni.ac.nz (G.M.N.); a.d.j.taylor@bath.ac.uk (A.D.J.T.)

**Keywords:** social prescribing, pharmacy, mental health, wellbeing, pharmacy technician, pharmacist

## Abstract

Social prescribing is increasingly viewed as a non-pharmacological option to address psychosocial consequences of social isolation, loneliness and bereavement; key contributors to poor mental health and wellbeing. Our study explored experiences and attitudes of pharmacists and pharmacy technicians to social prescribing in England, Scotland, and Wales, using an on-line survey. (Ethical approval, University of Bath, November 2017). The electronic survey was distributed to pharmacists registered with Royal Pharmaceutical Society local practice forum network groups in England, Scotland, and Wales, and pharmacy technicians via social media platforms. Data were analysed using descriptive statistics and free text by thematic analysis. One hundred and twenty respondents took part in the survey; (94.6% pharmacists and 5.4% pharmacy technicians). Responses indicated a lack of knowledge and experience with social prescribing; however, there was enthusiasm for pharmacists and the wider pharmacy team to be involved in local social prescribing pathways. Respondents believed they were well positioned within the community and consequently able to be involved in identifying individuals that may benefit. Barriers to involvement, included time, funding and training while enablers were pharmacist skills and the need within the community for social prescribing. There is a willingness in pharmacy, to be involved in social prescribing, however further research is required to enable the pharmacy team to be full participants in social prescribing pathways.

## 1. Introduction

Evidence suggests that socioeconomic factors can have the greatest effect on health and wellbeing, accounting for 40% of all influences on the individual [1]. Other factors include social isolation, bereavement, and debt [2]. This implies that clinical services are not always most appropriate for helping individuals whose ailment may not have a clinical cause and therefore dealing with the underlying cause may be more effective than providing clinical treatment. Additionally, general practitioners (GPs) in England reported spending 20% of their time dealing with non-health related matters such as relationship issues and housing [3], providing further evidence why dealing to with these factors via social prescribing (SP) has been viewed as an option to decrease GP’s time spent on non-medical issues.

Social prescribing is a non-medical model, which incorporates a patient and values centred, psychosocial approach to collaboratively support individuals to identify and address the detrimental social determinants of their current position and collaboratively plan a way to address these together. SP has been defined as “a pathway to refer clients to non-clinical services; linking clients to support from within the community to promote their wellbeing, to encourage social inclusion, to promote self-care where appropriate and to build resilience within the community and for the individual” [2].

The SP pathway is usually started by a referral from a GP or other healthcare professional, who refers an individual to a social prescribing coordinator (sometimes called a link worker), who then meets the person to complete an individual assessment and agree a suitable activity. Activities such as gardening, exercise, woodwork, knitting, art, or cycling—often provided by charities and the voluntary sector—have been included in social prescribing schemes [4]. There is also access to advice for debt management, housing and other social issues. The SP coordinator follows up the individual during their SP activity. The coordinator is often not a healthcare professional; typically they are someone with an interest in supporting the needs of vulnerable people in their community, with training in psychosocial skills and communication [5,6]. In some SP schemes, pharmacists in community and hospital settings have been involved in signposting to schemes, referral processes and providing a service/activity [7].

Due to the nature of SP, current research measures service demand, patient throughput and qualitative evaluation of the patient experience. A systematic review conducted by Kimberlee et al. (2017) [8], considered the evidence on the effectiveness of SP and there appeared to be a reduction in demand for National Health Service (NHS) services. However, the authors pointed out there was a large variation between the studies, and the extent of the effect on reductions on wider local NHS services, was dependent on the context and locality in which the study was based [9].

Recently there has been a call for more health professionals including pharmacists, to become involved in SP referrals [10]. Many pharmacists work in community or primary care settings, making them well positioned for a role in SP. However, prior to introducing SP pathways that include the pharmacy team, it is important to understand how much pharmacists already know about SP, their views on possible involvement, and their perceptions of potential barriers and enablers.

There is very little literature about pharmacist involvement in SP, so consequently the aim of this study was to explore the experiences and attitudes of pharmacists and pharmacy technicians in this area. The objectives were to:

(1) Explore the pharmacy team’s knowledge of social prescribing as a concept,
(2) Identify the pharmacy team’s awareness or experiences of local schemes
(3) Explore participant views on ways the pharmacy team can be involved,
(4) Identify barriers and enablers to the pharmacy team involvement.

## 2. Materials and Methods

A cross-sectional electronic survey was developed using Survey Monkey^®^ with question conceptualization arising from the findings of a previous SP study by the authors. This previous study included an interview cohort of six community pharmacists and explored their perceptions of SP and the place and role of community pharmacy involvement. (In Draft, 2019)

The face validity of our questionnaire was agreed by experts and the pilot was completed and commented on by a subset of 20 (community pharmacists in the Southwest of England in January 2018) of the potential participant sample to establish usability and readability. The recommended changes from the pilot feedback, were implemented. However, we did not complete principal components analysis or internal consistency. The final survey consisted of 35 questions, including demographic information (available in Supplementary Materials), and could be completed in approximately 15 min. There was a mixture of Likert scales; open-ended questions; yes/no questions; choice questions and open text questions. The latter question style allowed participants to express their attitudes to and experiences of SP. Although the survey was designed to be anonymous and protect confidentiality, participants could opt in to provide their email address to go into a prize draw to win a gift voucher, as a recognition and thank you for their time and participation. The final survey was distributed electronically via Royal Pharmaceutical Society (RPS) local practice forum networks in England, Scotland and Wales and by social media platforms for four weeks in January 2018.

### 2.1. Ethics

This study was approved by the University of Bath ethical review process In December 2017. National Research Ethics Service and Health Research Authority ethics approval were not required because this study did not fulfil their definition of a research project. The major ethical issue was the need to preserve confidentiality and anonymity of the participants and that study management conformed to the Data Protection Act and University of Bath regulations. Participants gave implied consent by opting in to take part in the survey.

### 2.2. Sampling and Recruitment

It was not possible to circulate the survey to all registered pharmacists in England, Scotland and Wales due to lack of access. In 2015, there were 55,000 registered pharmacists in the United Kingdom [11] and 44,667 were registered members of the Royal Pharmaceutical Society [12]. However, this latter figure also includes student and pre-registration pharmacists, and when these are removed, 53.1% of all registered pharmacists in the UK were members of the RPS in 2015. [12] This could affect the ability to translate findings across the wider pharmacy population.

### 2.3. Data Collection and Analysis

Data were collected using Survey Monkey and exported directly to Statistical Package for Social Sciences (SPSS) software version 23 for analysis. Data were ‘cleaned’ to ensure variables were correct and simple descriptive frequencies were run alongside chi-squared tests to look for correlations. However, no significant correlations were found. Free text was analysed using an inductive approach to thematic analysis as per Braun and Clarke [13].

## 3. Results

### 3.1. Demographics

One hundred and twenty respondents started the survey and 93 participants completed in full. However, data were analysed using responses to the question regardless of whether they completed the whole survey. For some questions, respondents were asked to select all that applied and thus not all responses match the number of participants. Eighty-eight (94.6%) were pharmacists and five (5.4%) were pharmacy technicians. There were 68 (73.1%) female respondents and 25 (26.9%) male respondents. Just over one-third (34.41%, *n* = 32) held a non-medical prescribing qualification and twenty-five of these respondents (26.9%) were currently prescribing in practice.

The following demographic figures illustrate the age of the respondent (Table 1), the number of years in practice (Table 2), and their professional sector of employment. (Table 3).

Participants could select as many care sectors as applicable. Of the 93 participants who completed the survey, thirteen worked in more than one care sector. Of those 13, five worked in a third care sector, illustrating the increasingly complex employment portfolios that pharmacy professionals hold. [14]

**Figure 1:**
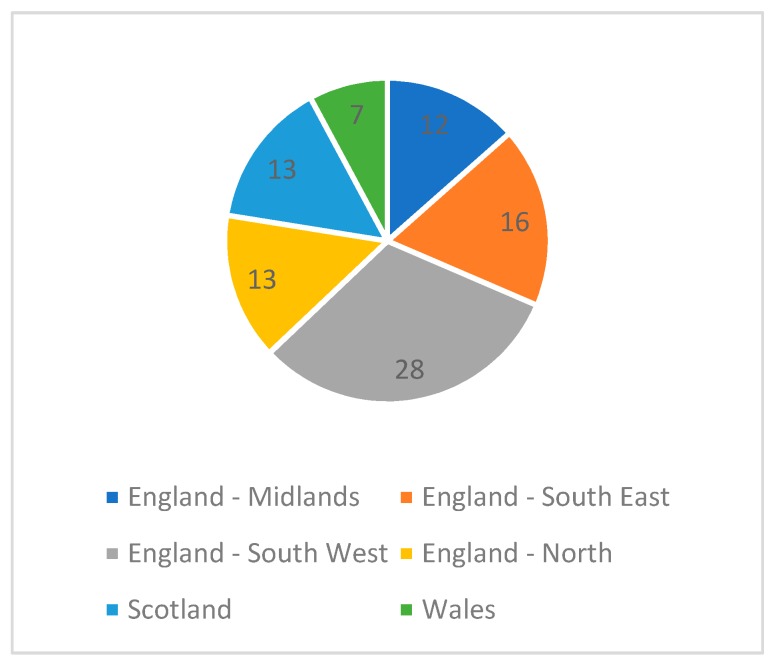
Main geographical area of practice.

Question 35 asked respondents to select which geographical region represented their main region of practice as highlighted in Figure 1 below. Eighty-nine respondents completed and 31 skipped this question. 77.5% (*n* = 69) respondents to this question mainly practiced in England, 14.6% (*n* = 13) in Scotland and 7.9% (*n*=7) in Wales.

### 3.2. Prior Knowledge and/or Experience of Social Prescribing

Forty-four participants (36.7%) had heard the term ‘social prescribing’ prior to completing the survey while 76 (63.3%) had not. This latter group included the five pharmacy technicians, all of whom had never heard the term before. Respondents who had previously heard the term mainly practiced in primary care and community pharmacy. Analysis of free text answers provided by respondents when asked about their personal understanding of what SP was, resulted in six key themes. These were:

- treatment without the use of medication (*n* = 15);
- treatment that is non-clinical or non-medical (*n* = 12);
- exercise on prescription/lifestyle changes (*n* = 4);
- a way of linking patients to services in the community to improve health, wellbeing, and/or social interaction (*n* = 4);
- treatment specialised for a specific need e.g., social isolation/increasing activity (*n* = 3);
- a way to refer patients to groups/organisations that provide social care (*n* = 3);
- emergency (A&E) attendance (*n* = 1); and 2 were not sure, but had heard the term before.

This is exemplified by the following quotes:

“My understanding is where (social) prescribing is for non-medicinal strategies, for example wellbeing, CBT, dietary, exercise, support.”

Respondent 23 (Pharmacist)

“Physical activity rather than a pill for every ill.”

Respondent 45 (Pharmacist)

Eighteen (39.1%) of those who were familiar with the term were aware of SP schemes in their local area, while 28 (60.9%) were not. Free text answers grouped into two main categories: those that knew of specific exercise or health related schemes (*n* = 11), and those who were just aware that schemes were available (*n* = 7).

Only six of 120 respondents had been directly involved in SP project and Table 4 illustrates the different schemes respondents were involved with.

### 3.3. The Appropriateness of the Name “Social Prescribing”

Our previous research (In Draft) suggests that healthcare professionals and the public felt ‘social prescribing’ was an inappropriate term as it linked the non-medical model to a more paternalistic medical model. When respondents were asked if they believed SP was an appropriate name for this process, forty-four (39.3%) agreed it was, but in contrast sixty-eight people (60.7%) believed it was inappropriate. The question went on to suggest alternative names for ‘Social Prescribing’ (see Table 5), which respondents could select or suggest their own.

Alternative names suggested by respondents (excepting one respondent who believed all options given were better than the term SP), were:

- Wellbeing intervention;
- Social activity programme;
- Social wellbeing scheme/programme;
- Wellbeing referral or wellbeing activity referral;
- Social activity prescribing;
- Health and wellbeing pathways.

### 3.4. Beliefs about Social Prescribing

Respondents were asked about their beliefs on social prescribing and the way in which it might benefit the recipient of this service. (See Table 6 later) Overall the views on SP were positive with 96.4% (*n* = 107) either agreeing or strongly agreeing that SP is a valid approach to healthcare. One hundred and five (94.6%) of respondents to this question believed SP addressed the emotional needs of participants and 98.2% (*n* = 109) believed it would help SP participants in general. Only three respondents (2.7%) disagreed and believed pharmacists did not have a role in SP and that it would not benefit individuals who took part in SP.

### 3.5. Who Should Be Involved?

Twenty-five (26%) respondents believed only healthcare professionals should be involved in SP, whereas the majority (74%, *n* = 71) believed SP should not be limited to healthcare professionals, and include a wider range of individuals and other professions.

*“HCP qualifications do not seem necessarily required for this type of intervention, it should be expanded to other people too, as then it has more likelihood of succeeding and being managed through larger networks.”*

Participant 13 (Pharmacist)

*“Any person should be able to refer a needy patient for help.”*

Participant 76 (Pharmacy Technician)

Of those who believed SP should only include healthcare professionals, the most common emergent theme from free text responses, was the need for the process to have some type of regulation to ensure those with real clinical needs had access to appropriate and timely clinical care. These also included ensuring individuals were referred to the most appropriate services based on their needs. Another theme was that healthcare professionals would have access to health information and the wider healthcare team of the patient; but non-health professionals would not have the same access. A few respondents worried about the cost to the wider NHS if those who were not professionally trained made inappropriate referrals to health services. It is important to note, that a few respondents believed that only healthcare professionals should be involved in certain aspects of SP, particularly the prescribing of medicines, but other areas would not exclusively need healthcare professionals. Participants felt that the training healthcare professionals received ensured the advice they delivered to potential SP participants was safe.

The respondents who indicated that SP was not just a role for healthcare professionals, believed that healthcare professional qualifications were not necessary, because in SP you did not need to be an expert to refer individual’s needing SP activities.

*“Some vulnerable people may not come into contact with healthcare professionals on a regular basis; however there may be others in the community with whom they have contact.”*

Participant 37 (Pharmacist)

The caveat to this was that anyone who held a referring role should have appropriate training. Respondents identified the following groups of people as being able to refer to a SP pathway: carers, peer group members, other staff working in health, teachers, social workers, and self-referral by the public. Members of these groups all had regular close contact with individuals who may benefit from SP. Within pharmacy, respondents suggested dispensers and counter assistants would be suitable for this role and this could also help to release pharmacist time.

#### 3.5.1. Pharmacist Involvement in Social Prescribing

Eight-six (89.6%) of respondents believed pharmacists should be involved in SP whereas 10 (10.4%) did not. For those who believed pharmacists should be involved in SP, the most common theme was the accessibility of pharmacists in the community with no need to book an appointment, and the strong relationships that develop with their patients. Others expressed this role should already be part of a pharmacist’s job as it is their duty to help patients, even if it does not directly involve medication. Other comments included that pharmacist involvement could reduce GP workload by minimizing unnecessary appointments; pharmacy was an important part of the healthcare team and importantly, that as pharmacists, they have seen that medication is not always the answer, and an alternative option was needed.

*“We can get to know patients over consultations for different care aspects, and may come to realise that drugs are not always the most appropriate therapy. As HCPs we should be signposting for all aspects of healthy lifestyle changes.”*

Participant 90, Pharmacist

For those who believed pharmacists should not be involved in SP, respondent comments included pharmacists already had enough to do and the lack of resources and funding to implement SP in community pharmacy was a barrier. Others shared that SP was not within the pharmacists’ remit and other professionals were better placed to be involved in SP.

*“Pharmacists are there to clinically check/provide/counsel patients on medicines. Although a nice idea (and something other healthcare professionals/volunteers are suitable placed to encourage/advise patients on this), this activity is not in the pharmacist remit.”*

Participant 24, Pharmacist

#### 3.5.2. Pharmacy Team Involvement

Overall, eighty respondents (83.3%) believed the entire pharmacy team should be involved; including 100% (*n* = 5) of pharmacy technicians and 81.8% (*n* = 72) of pharmacists. Free text analysis demonstrates the most common theme was the belief that anyone with a bit of training would be capable of being part of SP, especially if they had experience in patient interaction—as many pharmacy technicians do. Similarly, many also saw benefit in the whole pharmacy team working together to be involved in SP, where everyone would have a role that best suited their skills and specialties. Others believed this would help with time pressures that arise from increased pharmacist workload. Also identified was that counter assistants and other pharmacy team members often spent more time talking to patients and would be valuable in identifying people who would benefit from SP.

*“Suitably trained technicians would be just as capable in providing this service, in some respects they may well be better placed to do so, to free up the pharmacists time for clinical roles.”*

Participant 1, Pharmacy Technician

Conversely 16.7% (*n* = 16) of pharmacist respondents did not believe the entire pharmacy team should be involved, due to the belief that the pharmacist is the professional and has appropriate training and that information may be taken more seriously by the public, if it was offered by a pharmacist.

### 3.6. Pharmacy Involvement in SP Services

Only 9 (9.5%) respondents already referred people to SP services, while 90.5% (*n* = 86) did not. Of those who did not currently refer, sixty-two (72.1%) envisioned a future opportunity to refer patients to SP activities whereas 27.9% (*n* = 24) did not. Table 7 illustrates the number of respondents (*n* = 95) that would be willing to be involved in various steps of the SP pathway (respondents could tick more than one item).

Respondents felt more confident about being able to identify people who may benefit from SP and then referring to the SP team. There was high support for the delivery of a pharmacy-based SP service.

### 3.7. Barriers and Enablers

Respondents were asked to consider a number of different factors and select whether they perceived these as enablers or barriers to pharmacist and pharmacy team participation in SP. Findings from this question can be viewed in Table 8 below.

The top five enabling factors for SP as perceived by the respondents were:

(1) the need for SP in the local community (78.9%, *n* = 75)
(2) pharmacist skill in identifying potential individuals’ for SP (73.7%, *n* = 70)
(3) pharmacist desire to be involved in SP (70.6%, *n* = 67)
(4) evidence for the benefit of SP (67.4%, *n* + 64)
(5) availability of a consultation room (66.3%, *n* = 63)

The biggest barriers were lack of time for consultations (48.4%, *n* = 46) and the relative employment costs of pharmacists (48.4%, *n* = 46). Respondents were also invited to suggest perceived enablers or barriers they believed existed. Twenty-three people responded, and Table 9 illustrates their viewpoints.

### 3.8. Pharmacy Confidence in Being Part of a Social Prescribing Pathway

Fifty-two (55.9%) respondents felt either confident (*n* = 38) or very confident (*n* = 14) in identifying people who may benefit from SP, while 9.7% (*n* = 9) felt unconfident (*n* = 6) or very unconfident (*n* = 3) in identifying those who may benefit from SP. Thirty-two (34.4%) remained unsure. Fifty-eight (62.4%) respondents were confident in identifying individuals that may benefit from non-clinical services, while 37.6% (*n* = 35) were not. Importantly, only twenty-eight respondents (30.1%) knew where they could refer these individuals to, once identified. A possible solution to this is the provision and ongoing maintenance of an electronic database of services and/or a regularly updated and maintained website of SP activities in their local area, where individuals can be referred to. Eighty-seven (93.6%) respondents indicated this would be helpful and would want access to it.

Fifteen (16.1%) respondents were already supporting individuals’ in non-clinical activities as illustrated in Table 10 below.

### 3.9. Training

Overall, when respondents were asked to select what training they felt they needed, SP-specific training was highly represented, especially information around activities, and communication skills as seen in Table 11 below. ‘Other’ responses included: needing to see an evidence base for SP effectiveness; more practical training, for example what setting it would take place in, what current pharmacy services may have to be sacrificed, and training on how to have a more holistic view of healthcare. Ninety-three participants responded to this question and 27 decided not to.

## 4. Discussion

### 4.1. Limitations

This is a small study with a low response rate and the data is not generalizable, however the findings present a snapshot of current views and experiences of the pharmacy team on social prescribing. The survey link was emailed only to those who were registered with the RPS local practice forum network groups, with the potential for bias as it excluded non-RPS members. Members of the RPS may also hold special characteristics affecting generalizability. Although the survey asked potential respondents to participate even if they had not heard about social prescribing, it could indicate that lack of knowledge may also have deterred potential respondents from taking part and be partially responsible for the low response rate.

### 4.2. Implications for Including Pharmacy in Social Prescribing Pathways

This study demonstrated the views of pharmacists and pharmacy technicians around SP. Importantly most pharmacists and no pharmacy technicians had heard of SP prior to participating in this survey; however, there was a keenness to be involved once they knew more. This may be partially due to the emergence of pharmacist-led public health interventions within local communities by Healthy Living Pharmacies [15,16] who also promote an ethos of holistic health care.

The majority of respondents believed that SP was a valid approach to psychosocial healthcare and would benefit people who took part in it. Importantly, respondents believed the whole pharmacy team had a role to play in a social prescribing pathway and this would benefit their GP colleagues by reducing non-medical appointments. The greatest barriers were funding of the relevant pharmacy team members to take part and making times for consultations; but enablers were the skills they already possessed and the desire for pharmacy to be involved in local schemes. There is a need for dedicated funding for pharmacy involvement in social prescribing, a request which has also been highlighted by general practitioners. [17] Further research is needed to ascertain how SP could be implemented effectively in a pharmacy setting and the level of training needed.

Respondents were keen to be involved and believed pharmacy was an important component of asocial prescribing pathway, however they also identified the need for appropriate training and funding for the pharmacy workforce. Particularly, in identifying local schemes and appropriate individuals and the referral process, as well as the need for more evidence regarding the effectiveness of social prescribing. Addressing these training and organisational issues are important steps that need to be addressed, prior to inclusion of pharmacy in social prescribing pathways.

Evidence has demonstrated far-reaching outcomes from social prescribing for the individual, those around them, and for the public sector [18,19]. Importantly in 2018 social prescribing was identified as a leading option for supporting people with loneliness in the United Kingdom [20]. Starting with the inclusion of Heathy Living Pharmacies into local social prescribing pathways would seem to be the first step in this process as staff within these pharmacies have completed extra training in public health and mental health and wellbeing and are routinely involved in supporting people in their local communities. There is also a pre-requisite for such pharmacies to re-model their pharmacy to be patient friendly and welcoming and promoting a more holistic approach to health. Including the pharmacy team in social prescribing pathways would widen the ability to support people with psychosocial needs arising from non-medical determinants and reach people who are unable to access general practice health services.

## 5. Conclusions

Responses indicated a lack of knowledge and experience with social prescribing amongst the pharmacy team; however, there was enthusiasm for pharmacists and the wider pharmacy team to be involved in local social prescribing pathways. Respondents believed they were well positioned within the community and consequently able to be involved in identifying individuals that may benefit. Barriers to involvement, included time, funding and training, while enablers were pharmacist skills and the need within the community for social prescribing. There is a willingness in the pharmacy profession to be involved in social prescribing, however further research is required to enable pharmacy to be full participants in social prescribing pathways.

## Figures and Tables

**Table 1:** Age of Respondents.

Age (Years)	Respondents *n* = (%)
20–25	• 5 (5.4)
26–35	• 25 (26.9)
36–45	• 25 (26.9)
46–55	• 17 (18.3)
>56	• 21 (22.5)
**Total (*n*=)**	• **93 (100%)**

**Table 2:** Years Qualified

Years Qualified	Respondents *n* = (%)
0–5	• 14 (15.1)
6–10	• 15 (16.1)
11–15	• 16 (17.2)
16–20	• 7 (7.5)
>21	• 41 (44.1)
**Total (*n*=)**	• **93 (100%)**

**Table 3:** Sector of Employment.

Sector	Respondents
Hospital	• 37
Primary Care	• 23
Community health provider	• 1
Community pharmacy	• 36
Retired/not working	• 3
Industry	• 2
Prison	• 1
Health policy	• 4
Academia and education	• 4
**Total (*n*=)**	• **112**

**Table 4:** Respondent Roles in Social Prescribing.

Respondent Activity/Role	Social Prescribing Pathway
Supporting local schemes	Govan SHIP project Supporting the Local Care Network to develop their project in SE Lambeth, and providing information and helping to promote at CCG meetings
Specialist services	Specialist HIV Pharmacist Books on prescription issued following review of antidepressant therapy
Referral	Referring patients to local schemes or the local Patient Champion Choir for those with Parkinson’s and dementia
Prescribing	Prescribing certain literature, prescribing exercise

**Table 5:** Is ‘Social Prescribing’ the most appropriate name?

Alternative Name Suggestion	Respondents *n* =(%)
Social activity pathway	• 28 (40.6)
Community referrals	• 17 (24.6)
Social referral programme	• 14 (20.3)
Social intervention	• 2 (2.9)
Other	• 8 (11.6)
**Total (*n*=)**	• **69 (100%)**

**Table 6:** Beliefs about social prescribing (111 participants).

Belief	Agree or Strongly Agree *n* = (%)	Neither Agree or Disagree *n* = (%)	Disagree or Strongly Disagree *n* = (%)
I believe social prescribing could be a valid approach to healthcare	107 (96.4)	3 (2.70)	1 (0.90)
I believe social prescribing could address the social and emotional needs of those who take part.	105 (94.6)	6 (5.41)	0
I believe social prescribing could benefit individuals who take part in it.	109 (98.2)	2 (1.80)	0
I believe pharmacy and pharmacists could have a role in social prescribing.	95 (85.6)	13 (11.7)	3 (2.70)
I believe pharmacist involvement in social prescribing could benefit the individuals who take part	93 (83.8)	15 (13.51)	3 (2.70)

**Table 7:** Willingness to be involved in certain parts of the Social Prescribing pathway.

Acceptable Level of Involvement	Respondents (*n* =)
Identifying individuals suitable for SP	• 75
Delivering a pharmacy related SP service as appropriate	• 51
Introducing the concept of SP & referring to the SP coordinator	• 48
Monitoring the individual in their engagement with SP activities	• 31
Introducing the concept of SP to an individual and referring to GP	• 29
Being a SP coordinator and after completing a needs assessment, agreeing a SP plan	• 29
Other	• 14
I am not willing to be involved	• 4

**Table 8:** Perceptions of Enablers and Barriers for Pharmacy Involvement in Social Prescribing.

Barriers/Enablers	Strong Enabler *n* = (%)	Enabler *n* = (%)	Neither Enabler or Barrier *n* = (%)	Barrier *n* = (%)	Strong Barrier *n* = (%)	Total
Available space for consultation	30 (31.6)	33 (34.7)	16 (16.8)	15 (15.8)	1 (1.1)	95
Funding available	32 (33.7)	12 (12.6)	13 (13.7)	25 (26.3)	13 (13.7)	95
Pharmacist skill in detecting those that may benefit from SP	26 (27.4)	44 (46.3)	16 (16.8)	7 (7.4)	2 (2.1)	95
Need within the community for SP	35 (36.8)	40 (42.1)	17 (17.9)	2 (2.1)	1 (1.1)	95
Pharmacist desire to be involved in SP	26 (27.4)	41 (43.2)	13 (13.7)	14 (14.7)	1 (1.1)	95
Evidence of the benefit of SP	19 (20.0)	45 (47.4)	20 (21.1)	7 (7.4)	4 (4.2)	95
Available time for more consultations	27 (28.4)	13 (13.7)	9 (9.5)	34 (35.8)	12 (12.6)	95
Knowledge of current local SP pathways	28 (29.5)	22 (23.2)	14 (14.7)	26 (27.4)	5 (5.3)	95
Employment cost of pharmacists	12 (12.6)	15 (15.8)	22 (23.2)	33 (34.7)	13 (13.7)	95
Skill set of wider pharmacy team	18 (19.0)	35 (36.8)	19 (20.0)	19 (20.0)	4 (4.2)	95

**Table 9:** Respondent’s Perspectives of Barriers and Enablers to Pharmacy in Social Prescribing.

Enablers	Barriers
Funding	Funding/lack of sustainability
Time	Time
Knowledge of scheme	Lack of knowledge of schemes
Expertise	Lack of expertise
Regular contact with patients	Variation in patients
Perceived need by commissioners	Lack of support from senior members of staff
Collaborative working	Collaborative working with GP’s and activity
The pharmacy being seen as a respected voice and being easily accessible	Providers—particularly confidentiality
	Non-pharmacist managers
	Lack of confidence

**Table 10:** Respondent’s Involved in Non-clinical Activities.

Services	Responses *n* =
Weight management	4
Exercise	3
Smoking cessation	1
Patient champion and referral	1
Cancer services	1
Other services	1

**Table 11:** Perceived Training Needs for Involvement in Social Prescribing.

Training Need	Responses *n*=
What activities are available	83
Inclusion criteria for activities	81
Understanding more about social prescribing	72
Pharmacist roles in social prescribing	67
Condition specific information such as signs and symptoms	61
Communication skills	25
Other	7

## Supplementary Materials

The following are available online at https://www.mdpi.com/2226-4787/7/1/24/s1, Final Survey Questions.
