# Supplementary material for: Perceptions of Pharmacy Involvement in Social Prescribing Pathways in England, Scotland and Wales
Source: Pharmacy (Basel). 2019 Mar 4;7(1):24. doi: 10.3390/pharmacy7010024 (PMC6473812; doi:10.3390/pharmacy7010024)
Supplement: Supplementary file 1 [file pharmacy-07-00024-s001.pdf]

Introduction

Hello and welcome,

Thank you for opting to take part in this survey.

You have been asked to take part in this survey as you are a registered pharmacist in England, Scotland, or Wales and we are exploring your views on and experiences of social prescribing.

This survey is for all registered pharmacists, regardless of your prior knowledge and/or experience of social prescribing.

The aim of this study is to explore pharmacist knowledge and experience of social prescribing in England, Scotland and Wales. In particular, we are interested in:

- Pharmacist awareness of social prescribing as a concept
- Pharmacist experiences of local social prescribing schemes
- How and if pharmacists can be involved in social prescribing, and
- Barriers and enablers to pharmacist involvement.

This study has been approved by the ethical review process of the Department of Pharmacy and Pharmacology, University of Bath and is part of the Universitiy's Undergraduate Research Project scheme for 2017/2018

The responses to this survey will be kept anonymously, and your data will be held securely in a password protected folder hosted on a secure university server, where it will be retained for 5 years before being destroyed.

The results of this survey may contribute to the planning of future social prescribing services which include input from pharmacists.

This survey should take around 20 minutes to complete

As a thank you for taking part, there will be an option to enter a prize draw to win one £50; one £20; or one of two £10 gift vouchers. The winners will be drawn at random after survey closes and will be notified by email.

If you have any questions regarding the survey or would like any further information, please feel free to email Dr Denise Taylor on d.a.taylor@bath.ac.uk or Dr Andrea Taylor on a.d.j.taylor@bath.ac.uk

Your understanding of social prescribing

* 1. Had you heard of the term 'social prescribing' before this survey?

- Yes
- No

If you answered no, please skip to question 5

2. If yes, tell us what your understanding of the term is

3. Are you aware of any social prescribing schemes in your local area?

- Yes
- No

4. If yes, can you please provide examples of these schemes

* 5. Have you been directly involved in any social prescribing projects?

- Yes
- No

6. If yes, can you please tell us what your role and/or input was

Definitions about the social prescribing process

Social prescribing has been defined as "*a* ***pathway*** *to refer clients to* ***non-clinical services****, linking clients to support from* ***within the community*** *to promote their wellbeing, to encourage social inclusion, to promote self-care where appropriate and to build resilience within the community and for the individual*" -Social Prescribing in Bristol Working Group, 2012

The actual pathway is usually started by a referral from a GP or other healthcare professional, who refers an individual to a social prescribing coordinator (sometimes called a link worker), who then meets the person to complete an individual assessment and agree a suitable activity. Activities such as **gardening, exercise, knitting, art, or cycling -** which are often provided by charities and the voluntary sector **-** have been included in social prescribing schemes. The social prescribing coordinator will then follow up the individual during the course of their social prescribing activity. This coordinator is often not a health care professional, typically they are someone with an interest in supporting needs of vulnerable people in their community.

In some social prescribing schemes, pharmacists in community and hospital settings have been involved in signposting to schemes, referral processes and providing a service/activity.

After reading the definition of social prescribing, please respond to the following questions

* 7. Do you believe social prescribing is an appropriate term for this process

- ◯ Yes
- ◯ No

8. If answered no, which term would be more appropriate? Please select one term only

- ◯ Social referral programme
- ◯ Social activity pathway
- ◯ Community referrals
- ◯ Social intervention
- ◯ Other (please provide an alternative)

[ ]

Personal beliefs about social prescribing

* 9. Please indicate the extent to which you agree or disagree with the following statements:

	Strongly agree	Agree	Neither agree or disagree	Disagree	Strongly disagree
I believe social prescribing could be a valid approach to healthcare.	○	○	○	○	○
I believe social prescribing could address the social and emotional needs of those who take part.	○	○	○	○	○
I believe social prescribing could benefit individuals who take part in it.	○	○	○	○	○
I believe pharmacy and pharmacists could have a role in social prescribing.	○	○	○	○	○
I believe pharmacist involvement in social prescribing could benefit the individuals who take part.	○	○	○	○	○

Roles in social prescribing and pharmacy involvement

There are 6 possible roles in the social prescribing (SP) pathway where pharmacy could be involved in:

1. Identifying an individual suitable for SP.
2. Introducing the concept of SP to an individual & refer to the SP coordinator if they want to take part.
3. Introducing the concept of SP to an individual & refer to GP for SP consideration.
4. Being a SP coordinator and after completing a needs assessment, agree a SP plan and activities with the individual.
5. Monitoring the individual and their engagement with SP activity.
6. Delivering a pharmacy related SP service as appropriate e.g. MUR, weight reduction, smoking cessation.

GP's have a direct role in referring individuals to social prescribing schemes, but Pharmacists and other health care professionals could be involved.

* 10. Do you think social prescribing should be the role of health care professionals only? (Where a health care professional means a person involved in the provision of health care services to an individual patient)

○ yes

○ no

* 11. Please explain your answer

[ ]

* 12. Do you think pharmacists should be involved in social prescribing?

○ Yes

○ No

* 13. Please explain your answer

[ ]

* 14. Do you think the whole pharmacy team (rather than pharmacists alone) should be involved in social prescribing?

◯ Yes

◯ No

* 15. Please explain your choice

[ ]

* 16. Do you currently refer people to social prescribing services?

- ○ Yes
- ○ No

17. If you answered no in your current role, do you see a future opportunity to refer patients to social prescribing activities?

- ○ Yes
- ○ No

* 18. What steps of the social prescribing pathway would you be willing to be involved in? (please select all that apply)

- ☐ Identifying individuals who are suitable for social prescribing
- ☐ Introducing the concept of social prescribing to an individual and referring the individual to a social prescribing coordinator if they want to take part
- ☐ Introducing the concept of social prescribing to an individual and referring them to a GP for social prescribing consideration
- ☐ Being a social prescribing coordinator and after completing a needs assessment, agree a social prescribing plan and activities with the individual
- ☐ Monitoring the individual and their engagement with social prescribing activities
- ☐ Delivering a pharmacy related social prescribing service as appropriate
- ☐ I am not willing to be involved
- ☐ Other (please specify)

* 19. The list below looks at different factors that may facilitate (enable) or be a barrier to social prescribing by pharmacists. From your own perspective, please select the option that best describes your view of each factor

	Strong enabler	Enabler	Neither enabler or barrier	Barrier	Strong barrier
Available space for consultation	◯	◯	◯	◯	◯
Funding available	◯	◯	◯	◯	◯
Pharmacist skill in detecting those that may benefit from social prescribing	◯	◯	◯	◯	◯
Need within the community for social prescribing	◯	◯	◯	◯	◯
Pharmacist desire to be involved in social prescribing	◯	◯	◯	◯	◯
Evidence of the benefit of social prescribing	◯	◯	◯	◯	◯
Available time for more consultations	◯	◯	◯	◯	◯
Knowledge of social prescribing pathways already in place	◯	◯	◯	◯	◯
Employment cost of pharmacists	◯	◯	◯	◯	◯
Skill set of wider pharmacy team	◯	◯	◯	◯	◯

20. From the above list, if there are factors missing that you believe could enable or be a barrier to pharmacist involvement in social prescribing, please specify it below.

Enabler ____________________

Barrier ____________________

Signposting to social prescribing

For pharmacists to be involved more directly in social prescribing they need to be in an appropriate setting and be able to identify individuals who would benefit from these schemes.

* 21. How confident are you, that you could identify people who may benefit from social prescribing?

Very confident	Confident	Not sure	Unconfident	Very unconfident
○	○	○	○	○

* 22. In your current role, do you feel confident to identify people who would benefit from non-clinical services?

- ○ Yes
- ○ No

* 23. In your current role, do you know where you could refer people to, if you felt they needed and would benefit from non-clinical services?

- ○ Yes
- ○ No

* 24. Would you find it helpful to have an updating list of appropriate services available for referral? (For example a regularly maintained and updated website for social prescribing activities in your area)

- ○ Yes
- ○ No

* 25. Do you currently support people who are involved in non-clinical activities e.g exercise or weight loss prescriptions?

- ○ Yes
- ○ No

26. If answered yes, Please list the services you are involved in

[ ]

Training or education to support the pharmacist role in social prescribing

* 27. If you were to be more involved in social prescribing, what training would you need for that role? (please select all that apply)

- [ ] Understanding more about social prescribing
- [ ] Pharmacist roles in social prescribing pathway
- [ ] What activities are available
- [ ] Inclusion criteria for the activities
- [ ] Condition specific information such as signs and symptoms
- [ ] Communication skills
- [ ] Other (please specify)

And finally, about you:

To help aid in the analysis of these results there are just a few more questions about yourself

* 28. Years qualified:

- 0-5;
- 6-10;
- 11-15;
- 16-20;
- >21

* 29. Age:

- 20-25;
- 26-35;
- 36-45;
- 46-55;
- >56

* 30. Gender:

- Male
- Female
- Prefer not to say

* 31. Which sector(s) do you currently practice in? (Tick all that apply)

- [ ] Hospital - District General
- [ ] Hospital - Tertiary care
- [ ] Primary care - GP Surgery
- [ ] Primary care - Prescribing advisor
- [ ] Primary Care - Clinical commissioning
- [ ] Community Pharmacy – Large chain (more than 50)
- [ ] Community Pharmacy – medium chain (11-50)
- [ ] Community Pharmacy – Small chain (6-10)
- [ ] Community Pharmacy – Independent (less than 5)
- [ ] Community Pharmacy - GP surgery
- [ ] Community health provider - Prison
- [ ] Community health provider - other
- [ ] Other (please specify)

* 32. Do you currently hold a non-medical prescribing qualification?

- ○ Yes
- ○ No

* 33. If yes, do you currently prescribe

- ○ Yes
- ○ No

34. Where do you currently practice?

- [ ] England - Greater London
- [ ] England - South East
- [ ] England - South West
- [ ] England - West Midlands
- [ ] England - North West
- [ ] England - North East
- [ ] England - Yorkshire and the Humber
- [ ] England - East Midlands
- [ ] England - East of England
- [ ] Scotland - Edinburgh and the Lothians
- [ ] Scotland - South
- [ ] Scotland - Glasgow and the Clyde
- [ ] Scotland - Central
- [ ] Scotland - Argyll
- [ ] Scotland - Northeast
- [ ] Scotland - The Highlands
- [ ] Scotland - Skye and the Western Isles
- [ ] Scotland - Orkney and Shetland
- [ ] Wales - North West
- [ ] Wales - North East
- [ ] Wales - Mid Wales
- [ ] Wales - South West
- [ ] Wales - South East

Ending

Thank you for taking the time to complete this survey

35. If you would like to be entered into the prize draw please provide your name and email address below

Name

Email Address

36. If you would like to be informed of the findings, please provide your name and email address below

Name

Email Address

Please note we will destroy all names and email addresses once the winners have been drawn and contacted.
